# Efficient generation of single domain antibodies with high affinities and enhanced thermal stabilities

**DOI:** 10.1038/s41598-017-06277-x

**Published:** 2017-07-19

**Authors:** Naoya Shinozaki, Ryuji Hashimoto, Kiichi Fukui, Susumu Uchiyama

**Affiliations:** 1 Modality Research Laboratories, R&D Division, Daiichi Sankyo Co., Ltd., Shinagawa R&D Center, 1-2-58 Hiromachi, Shinagawa-ku Tokyo, 140-8710 Japan; 2 Graduate School of Pharmaceutical Sciences, Osaka University, 1-6 Yamadaoka, Suita Osaka, 565-0871 Japan; 3 Department of Biotechnology, Graduate School of Engineering, Osaka University, 2-1 Yamadaoka, Suita Osaka, 565-0871 Japan; 4 Okazaki Institute for Integrative Bioscience, National Institutes of Natural Sciences, 5-1 Higashiyama, Myodaiji, Okazaki Aichi, 444-8787 Japan

## Abstract

Single domain antibodies (sdAbs), made of natural single variable regions of camelid or cartilaginous fish antibodies, or unpaired variable regions of mouse or human IgGs, are some of the more promising biologic modalities. However, such conventional sdAbs have difficulties of either using unwieldy animals for immunization or having high affinity deficiencies. Herein, we offer a versatile method to generate rabbit variable domain of heavy chain (rVH) derived sdAbs with high affinities (*K*_D_ values of single digit nM or less) and enhanced thermal stabilities (equal to or even higher than those of camelid derived sdAbs). It was found that a variety of rVH binders, including those with high affinities, were efficiently acquired using an rVH-displaying phage library produced at a low temperature of 16 °C. By a simple method to introduce an additional disulfide bond, their unfolding temperatures were increased by more than 20 °C without severe loss of binding affinity. Differential scanning calorimetry analysis suggested that this highly efficient thermal stabilization was mainly attributed to the entropic contribution and unique thermodynamic character of the rVHs.

## Introduction

Antibodies are being used for various therapeutic applications due to their high target specificity and biological functions. Most of them have been developed based on the robust natural format of human or humanized IgG. Fragment antibodies such as Fab, single chain fragment variable (scFv), and single domain antibody (sdAb) have also been utilized as promising modalities to create next-generation antibodies, like bi- or multi-specific antibodies. Moreover, such fragment antibodies are expected to be an imaging reagent because their smaller molecular size enables high tissue penetration and rapid elimination from the body.

There are many reports on sdAbs composed of natural single heavy chain antibodies from camelids (VHH), and several reports on those of cartilaginous fishes (IgNAR)^1, 2^. As more available sources, variable regions of mouse or human antibodies have been employed for the creation of sdAbs^3–8^, however, these sdAbs showed lower binding affinities than those of IgG or VHH. The rabbit is another widely used animal for antibody acquisition. It is well known that rabbit IgGs generally show higher binding affinities than mouse IgGs^9, 10^, nevertheless, there are only a few reports regarding their variable regions. We hypothesized that rabbit variable regions may have the potential to be modified to the sdAb format.

At the beginning of this study, we hypothesized that rabbit variable domain of heavy chain (rVH) has the possibility of showing a higher binding affinity than rabbit variable domain of light chain (rVL), because rVH tends to have a longer CDR3 than rVL^11, 12^, which may create a larger diversity, wider binding surface area and higher specificity to various antigens. The very limited germ-line gene segment of rVH^9, 11, 12^ is also considered to be advantageous because its highly homologous framework would enable it to share various engineering benefits, such as humanization^9^.

Next, we considered the stabilizing effect of the VH-VL interaction to rVH^13–15^. To acquire rVH binders from immunized rabbits using a phage display system, rVHs separated from rVLs have to be displayed properly on the phage. The temperature at which phages are produced in *Escherichia coli* (*E. coli*) is presumed to be the key to achieving this. Previously, an attempt was made to acquire rVH binders using an rVH-displaying phage library produced at 25 °C^16^, which is lower than the conventional temperature for rabbit scFv-displaying phages (30 °C or 37 °C)^17–19^, resulting in obtainment of only weak binders. Thus, in this study, the temperature was further lowered to investigate its impact on the rVH display level and resulting acquisition of rVH binders.

In order to utilize rVHs for various applications, high thermal stability would be one of the most desired properties. There are many papers that shows improvement of physicochemical properties by phage display method. In such approaches, mutations are sometimes involved at or near the CDRs^20, 21^. To avoid the risk of affinity change by such mutations, we considered the enhancement of thermal stabilities of rVHs by introducing covalent bond into the fixed position of deep inside the framework. Other groups reported introduction of an additional (artificial) disulfide bond at the residues 54 and 78 of camelid VHH (IMGT numbering)^22–24^. Most of the VHHs were successfully stabilized by the disulfide bond with relatively small negative effect on the binding affinity to the antigen, while some VHHs lost their antigen binding abilities^24^. Therefore, we thought it intriguing to investigate the impact of the disulfide bond on the thermal stabilities and affinities of rVHs, and to learn what kind of factors control them.

In this study, we attempted to acquire a wide variety of rVHs against tumor antigens HER2 and HER3 through phage production at temperatures lower than 25 °C. The obtained rVHs were then characterized by antigen binding affinity and thermal stability. Finally, the new disulfide bond was introduced into obtained rVHs and its impact on the thermal stabilities and affinities was examined. We provide a new platform to generate potent and highly stable rVH derived sdAbs for various applications, including therapeutic uses.

## Results

### Phage production and acquisition of antigen specific rVHs

**Figure 1:**
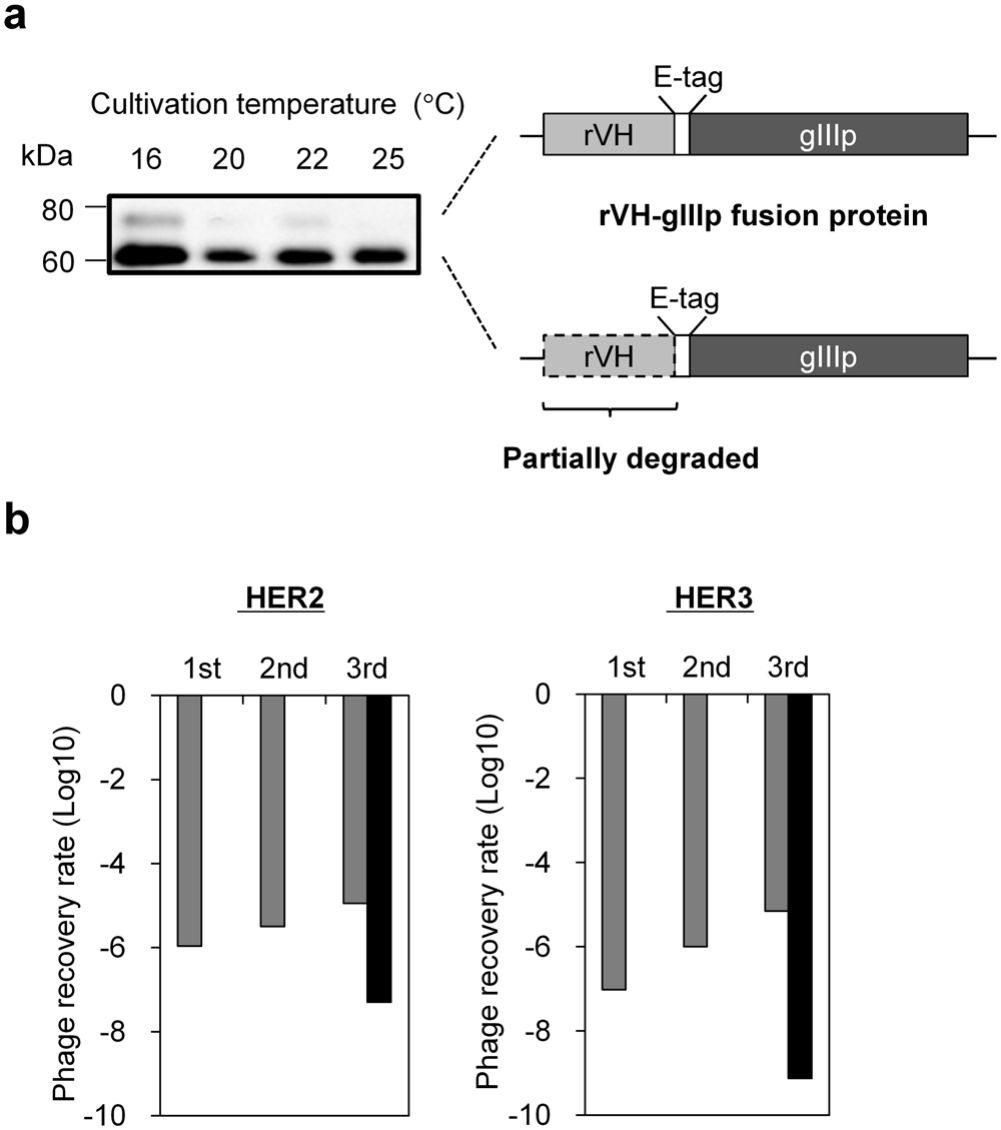
Panning against antigens with rVH-displaying phage. (**a**) Display level of rVHs on the phage produced at various temperatures. The phages were produced at 16, 20, 22 and 25 °C and subjected to WB (1.0 × 10^10^ virions per well). The rVH-gIIIp fusion protein was detected by anti-E-tag antibody and the amount of detected rVH-gIIIp fusion protein was correlated to the display level of rVHs. Fusion protein, which lacks an rVH portion, was also detected as a below band of the intact fusion protein. (**b**) Phage recovery rates after panning against HER2 or HER3. Gray bars indicate phage recovery rates (ratio of output to input phage) after each round of panning and black bars indicate phage recovery rates after the third round of panning without antigen.

Our rVH binder acquisition process is summarized in Supplementary Figure S1. Briefly, we immunized rabbits with HER2 and HER3 antigens and confirmed the increased antibody titers of these antigens in the immunized rabbits (Supplementary Fig. S2). We used a total of 1.9 × 10^8^ spleen and lymph node cells to amplify VH genes from these rabbits. rVH genes were amplified with designed primers (Supplementary Table S1) and inserted into phagemid vector. A total of 8.0 × 10^8^ transformants were obtained and it was expected that they would cover a wide variety of input rVH genes. For rVH-displayed phage production, we first cultivated the obtained transformants at several temperatures to investigate which temperature maximizes rVH display level. As shown in Fig. 1a, cultivation at 16 °C gave the strongest band intensity corresponding to rVH-gIIIp (gIII coat protein of M13 bacteriophage) fusion proteins, which are indicative of rVHs being displayed on the phage (Fig. 1a, *upper band*). The band become weaker as the cultivation temperature increased to 20 or 22 °C and was not detected at 25 °C. Thus, we adopted 16 °C as the cultivation temperature to produce an rVH-displaying phage library. The lower bands shown in Fig. 1a were considered to be impurities of rVH-gIIIp fusion proteins lacking the rVH region because their molecular weights (about 15 kDa) were consistent with the difference of molecular weight between the upper and lower bands. This consideration was supported by the fact that such an intense band was not observed for the control phage VCSM13, which is composed of only native gIIIp (Supplementary Fig. S3).

**Figure 2:**
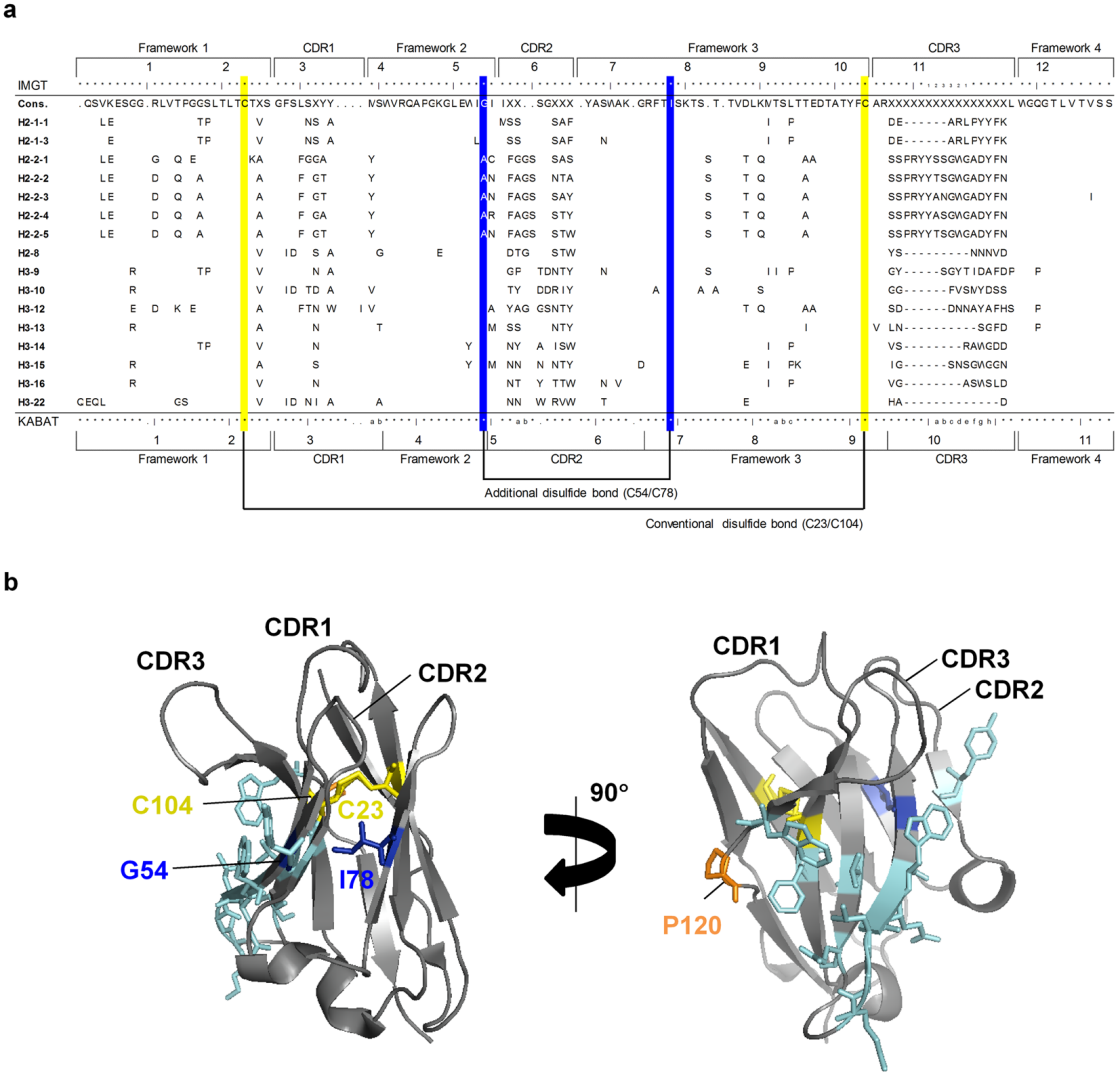
Primary and tertiary structures of rVHs (**a**) Amino acid sequences of the obtained 16 rVHs and their consensus (Cons.) sequences are aligned according to IMGT and KABAT numbering. For each obtained rVH, only amino acids different from consensus sequences are indicated. The Cys positions for C23-C104 and C54-C78 are linked with black lines and highlighted by yellow- and blue-filled frames, respectively. (**b**) The residues in the VL interacting surface (cyan or orange), a pair of Cys residues forming C23-C104 (yellow), and residues mutated to Cys for C54-C78 (blue) are indicated in the rVH structure (PDB ID: 4HBC). Figures were generated with PyMOL (The PyMOL Molecular Graphics System, 1.7.2.1 Schrodinger, LLC).

Starting from the prepared rVH phage library, phage recovery rates (numbers of output phage/input phage) increased step-wisely after every round of panning with each antigen (Fig. 1b). In the third round, remarkable differences in the phage recovery rates were confirmed between the panning with and without antigens. These results indicated that antigen binding rVHs were concentrated from a vast number of library clones. After three rounds of panning, about 300 output clones were screened for their binding abilities to antigens by Enzyme-Linked ImmunoSorbent Assay (ELISA), and 55 and 125 hit clones were obtained for HER2 and HER3 (Supplementary Fig. S1), respectively. These hit clones were subjected to sequence analysis and assessment of concentration-dependent binding to antigens (Supplementary Fig. S4). Non-specific binders were eliminated by counter screening for bovine serum albumin (BSA). Finally, eight rVHs were obtained respectively for both HER2 and HER3 (whose names start with H2 and H3 as Fig. 2a).

### Physico-chemical properties of antigen specific rVHs

We selected 5 hit clones from 16 clones in Supplementary Figure S4, which are two highly efficient ELISA binders (H2-2-2 and H3-15), moderate binder (H2-1-1) and two somewhat weak binders (H2-8 and H3-9), in order to assess the relationship between ELISA results and dissociation constants from SPR. We first tried to prepare these rVHs using *E. coli* and four rVHs except H2-1-1 could be obtained for enough amount to conduct physicochemical analysis. H2-1-1 were prepared using mammalian cell for physicochemical analysis. The purities of prepared rVHs were confirmed by SDS-PAGE analysis (Supplementary Fig. S5). rVHs commonly possess a disulfide bond between residues C23 and C104 (C23-C104) and prepared rVHs showed the formation of C23-C104 in MS analysis (Supplementary Table S2). We confirmed by SEC-MALS analysis that all rVHs were detected as the single peak around 15 kDa (Table 1), indicative of monomeric and mono-disperse states for the rVHs. We measured the binding affinities of these rVHs to respective antigens by surface plasmon resonance (SPR) analysis. H2-2-2 and H3-15 showed *K*_D_ values in a sub-nanomolar range; 0.4 nM for H2-2-2 and 0.8 nM for H3-15 (Table 1). *K*_D_ values of other rVHs (H2-1-1, H2-8 and H3-9) were in the range of from two to three-digit nM. Regarding the thermal stability, unfolding temperature (*T*_peak_) was determined by differential scanning calorimetry (DSC) analysis (Table 1). H2-1-1 and H2-2-2 showed lower *T*_peak_ values (47.9 and 37.7 °C, respectively) than those of H2-8, H3-9 and H3-15 (80.2, 66.9 and 61.9 °C, respectively). We plotted *T*_peak_ value and the purification yield in the *E. coli* expression system, and good correlation was observed between them (R² = 0.94, Supplementary Fig. S6).

**Table 1:** Characterizations of antigen specific rVHs.

		SPR			DSC		SEC-MALS	
		*k*_on_ (M^−1^ s^−1^)	*k*_off_ (s^−1^)	*K*_D_ (nM)	*T*_peak_ (°C)	∆*T*_peak_ (°C)	MW_cal_ (kDa)	MW_exp_ (kDa)
H2-1-1	WT	7.3 × 10^4^	1.6 × 10^−3^	22	47.9		15.3	17.0 ± 0.7
	Mutant	1.5 × 10^5^	1.4 × 10^−2^	92	78.2	30.3		
H2-2-2	WT	8.2 × 10^5^	3.3 × 10^−4^	0.4	37.7		16.0	18.9 ± 1.7
	Mutant	6.1 × 10^5^	1.6 × 10^−3^	2.7	60.4	22.7		
H2-8	WT	—	—	390^a^	80.2		14.9	13.3 ± 0.5
	Mutant	—	—	870^a^	100.8	20.6		
H3-9	WT	—	—	190^a^	66.9		15.5	13.9 ± 0.6
	Mutant	—	—	>5000^b^	90.3	23.4		
H3-15	WT	2.6 × 10^6^	2.0 × 10^−3^	0.8	61.9		15.3	15.6 ± 1.2
	Mutant	1.2 × 10^6^	2.5 × 10^−3^	2.0	81.8	19.9		

MW_cal_: Molecular weight (MW) calculated from amino acid composition.

MW_exp_: Experimentally obtained MW.

^a^For H2-8 and H3-9, *K*_D_ values were determined by steady–state affinity analysis.

^b^ *K*_D_ value for the C54-C78 mutant of H3-9 could not be determined up to 5 µM.

### Introduction of additional disulfide bond to rVHs

Aiming for thermal stability enhancements of the rVHs, we considered the introduction of an additional disulfide bond between residues 54 and 78 (Gly/Ala and Ile, blue-colored in Fig. 2a and b) based on the previous successful results in VHHs^22–24^. Because Cys is a hydrophobic amino acid, the residues of Cys mutations should be buried in a structure so as not to alter the hydrophobicity of the structure’s surface. In fact, accessible surface areas (ASAs) of residues mutated to Cys were less than 20% in the previous studies that reported the introduction of artificial disulfide bonds^22^. In order to estimate the ASA of residues for Cys mutation in the rVHs, we constructed model structures of five obtained rVHs using rabbit antibody structures that were available from a protein data bank (PDB) as a template (Supplementary Table S3). The estimated ASAs of the model structures were less than 10% (Supplementary Table S4), indicating that these residues were buried deep inside in the model structures. In order to investigate if the disulfide bond between C54 and C78 (C54-C78) would invoke structural alteration, we calculated the root mean square deviation (RMSD) between each model structure of rVHs with and without C54-C78. The Cα RMSDs of the five rVHs showed values similar to that of VHH compared to the α-subunit of human chorionic gonadotropin (VHH_hCG_, PBD ID: 1HCV). Because VHH_hCG_ was thermally stabilized by C54-C78 without loss of binding activity^22^, the introduction of C54-C78 was unlikely to have a negative impact on the rVHs’ binding affinities to antigens. Based on these considerations, in this study we attempted to introduce an additional disulfide bond into the wild type rVHs by Cys mutation of residues 54 and 78.

The rVHs whose residues 54 and 78 were mutated to Cys (C54-C78 mutant) were prepared by the same method as wild type rVHs and those purities were confirmed by SDS-PAGE analysis (Supplementary Fig. S5). It was not clearly observed that purification yields were improved by disulfide bond introduction under our preparation conditions using *E. coli*. In the case of H2-1-1, which was prepared using mammalian cell, purification yield of its C54-C78 mutant was increased to 3- or 7-fold compared with wild type. The formation of C54-C78 was experimentally confirmed by MS analysis after chymotrypsin digestion (Supplementary Table S4). Chymotrypsin digestion of mutant rVHs produced peptide fragments linked with C54-C78 or C23-C104 as expected. Neither undesired peptide fragments linked with other combinations of disulfide bond nor those with free Cys were detected. These results indicated that both of the two disulfide bonds were correctly formed as we had designed.

### Physico-chemical properties of C54-C78 mutant rVHs

**Figure 3:**
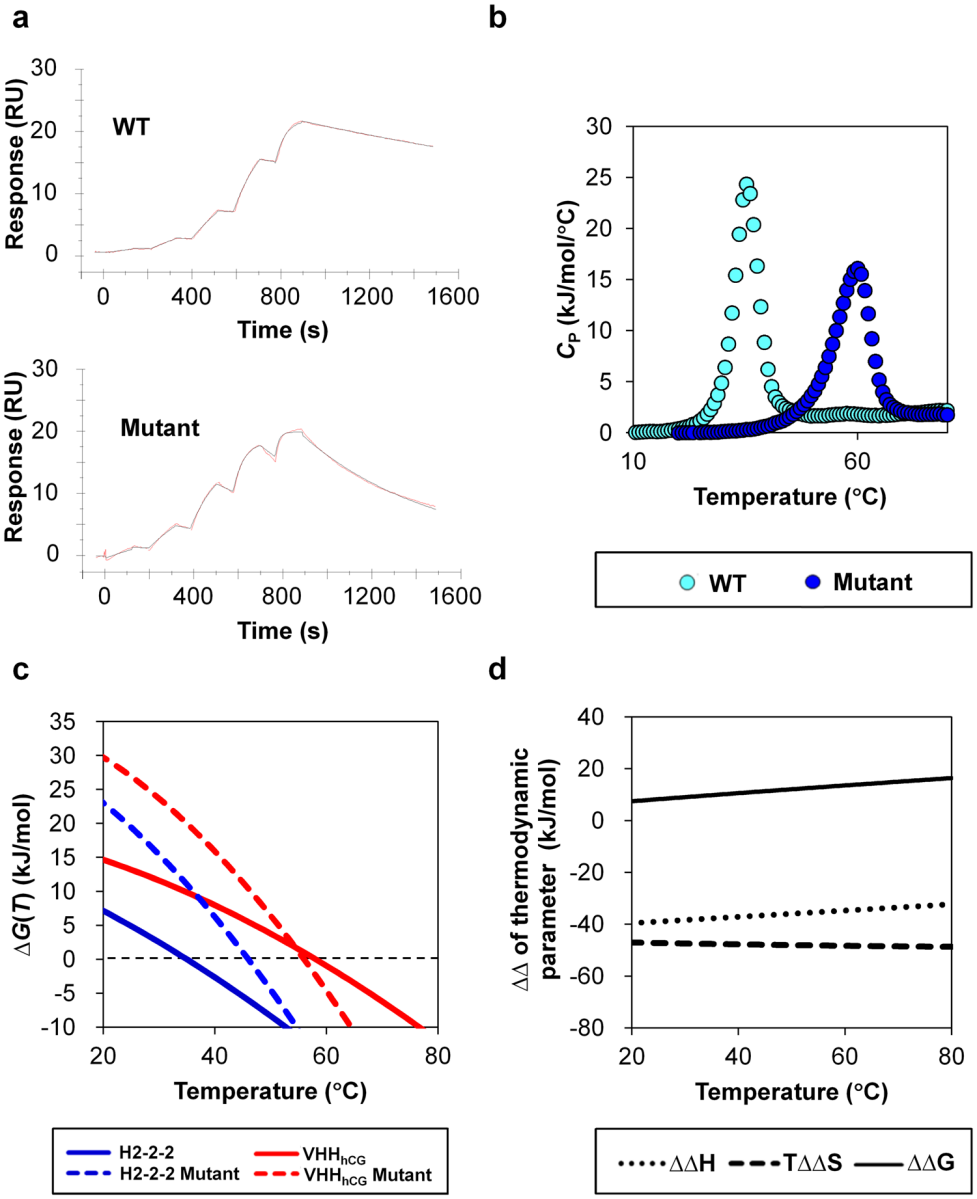
Physico-chemical properties of H2-2-2 and its C54-C78 mutant. (**a**) Observed sensorgrams (red) and fitting curves for single cycle kinetics (black) in the SPR analysis for H2-2-2. Data were collected at concentrations of 0.11, 0.37, 1.1, 3.3, 10 nM for H2-2-2 and 1.2, 3.7, 11, 33, 100 nM for its mutant. (**b**) The thermal unfolding curve of H2-2-2 (light blue) and its C54-C78 mutant (blue) in the DSC analysis. (**c**) The free energy change of H2-2-2 (blue) and VHH_hCG_ (red). Solid lines indicate those of wild types and dashed lines indicate those of C54-C78 mutant. (**d**) The differences in the thermodynamic parameter change between H-2-2-2 and its C54-C78 mutant. The differences in the change of free energy (ΔΔ*G*), enthalpy (ΔΔ*H*) and entropic term (*T*ΔΔ*S*) are indicated as the solid line, dashed line and dotted line, respectively.

The antigen binding affinities of C54-C78 mutant rVHs were evaluated by SPR. Their *K*_D_ values, except for H3-9, were within several-fold of their respective wild type counterparts (Fig. 3a and Table 1). The affinities of rVHs in Fig. 3a (H2–2–2 and its C54-C78 mutant) were also evaluated with BioLayer Interferometry method (BLI method). Using BLI method, which is completely different biosensor system from SPR, we could obtain similar fold change in *K*_D_ values as SPR between H2-2-2 and its mutant (6.3 fold for BLI, 6.8 fold for SPR, Supplementary Fig. S7). The thermal stabilities of C54-C78 mutants were evaluated (Table 1). The *T*_peak_ values of all mutants were more than 20 °C higher than those of corresponding wild type rVHs, and remarkably, such *T*_peak_ increases were much larger than those of previous results by VHHs^22–24^. rVHs can be highly thermally stabilized by C54-C78 introduction without severe loss of binding affinities.

In order to obtain information about the major factors that contributed to the high thermal stabilization mentioned above, the thermodynamic parameters and *T*_m_, at which the Gibbs free energy change (Δ*G*) becomes zero, were determined for H2-2-2 and its C54-C78 mutant by DSC (Fig. 3b and Table 2). In comparison, those of VHH_hCG_ were estimated based on the published data^22^ (Table 2). With the obtained thermodynamic parameters and *T*_m_, Δ*G* values at each temperature (Δ*G*(*T*)s) were illustrated in Fig. 3c. The introduction of C54-C78 increased Δ*G* of mutant of H2-2-2 and VHH_hGC_ to the same extent at *T*_m_ of wild type (${T}_{{\rm{m}}}^{{\rm{W}}}$). As for *T*_m_, the *T*_m_ increase (Δ*T*_m_) of H2-2-2 was more than 20 °C (from 35.0 °C to 58.1 °C), while that of VHH_hCG_ was 10 °C (from 46.0 °C to 56.0 °C). Comparing Δ*G*(*T*) curves of mutants and wild types, C54-C78 introduction did not have large impact on the shape of the Δ*G*(*T*) curve itself. Regarding the enthalpy and entropy change accompanied by the thermal unfolding (Δ*H* and Δ*S*), the mutant of H2-2-2 showed the values of 162.2 kJ/mol and 489.7 J/mol/K, respectively. These values were less than half those of mutant of VHH_hCG_ (369.2 kJ/mol and 1121.7 J/mol/K, respectively). In terms of the influence of the C54-C78 introduction on the Δ*H* and Δ*S*, compared to their wild types both Δ*H* and Δ*S* were slightly decreased for the mutant of H2-2-2 while were increased for the mutant of VHH_hCG_. The change in the heat capacity (Δ*C*_P_) of the mutant of H2-2-2 was 1.8 kJ/mol/K, which was one-third that of the mutant of VHH_hCG_. The C54-C78 introduction led to the slight increase in Δ*C*_P_ of both mutants of H2-2-2 and VHH_hCG_ compared to their wild types. Figure 3d shows the differences in the change of enthalpy, entropic term, and free energy (ΔΔ*H*, *T*ΔΔ*S* and ΔΔ*G*) between mutant and wild type of H2-2-2 at various temperatures. ΔΔ*H* was negative but largely compensated by *T*ΔΔ*S*, resulting in a positive ΔΔ*G* at all indicated temperatures.

**Table 2:** Thermodynamic parameters of H2-2-2 and VHH_hCG_.

		*T*_m_ (°C)	∆*H* (kJ/mol)	∆*S* (J/mol/K)	∆*C*_P_ (kJ/mol/K)
H2-2-2	WT	35.1	161.4 ± 1.9	523.8 ± 6.0	1.5 ± 0.1
	Mutant	58.1	158.6 ± 3.2	478.8 ± 9.6	1.7 ± 0.1
VHH_hCG_	WT	46.0	346.8 ± 5.4	1086.6 ± 16.9	4.8 ± 0.4
	Mutant	56.0	369.2 ± 9.4	1121.7 ± 28.6	5.2 ± 0.4

For VHH_hCG_, *T*_m_ and ∆*C*_P_ were quoted from ref. 22 and other thermodynamic parameters were calculated using published data^22^.

## Discussion

In this study, rVHs were shown to have the potential for specific binding to antigens with sub-nanomolar *K*_D_ values. Based on our knowledge, this is the first report to obtain such high affinity binders composed of an unpaired variable region^3–8^. As correlation was observed for representative five rVHs between binding efficiencies of ELISA (Supplementary Fig. S4) and dissociation constants of SPR (Table 1), we consider that other ELISA binders such as H2-2-1 or H3-14, which are as efficient as H2-2-2 and H3-15, could also have smaller dissociation constants as those of H2-2-2 and H3-15. Physical stability of therapeutic proteins in solution is governed mainly by the combination of conformational stability that corresponds to the free energy difference between native and denatured states and colloidal stability that reflects the dispersion state of the protein molecules^25, 26^. Upon considering the acquisition of the rVHs, their reduction in conformational stabilities was presumed because they lacked partner VLs^13–15^. In fact, the difference in the free energy between scFv and unpaired VH was calculated to be 9.0 kJ/mol from the denaturation curve in the previous report^13^. This large decrease in the stability elicited our concern that phage production at high temperatures would cause inefficient display of rVHs on the phage. The phage produced at 25 °C, which was used in the previous study^16^, did not show a detectable level of rVH display (Fig. 1a). While, the rVH display level was enhanced by lowering temperature and maximized at 16 °C. Using a rVH-displaying phage library produced at 16 °C, we could obtain a variety of HER2 and HER3 binders. Some of rVHs were poorly produced in *E. coli* and thermally unstable (Supplementary Fig. S6). This result implied that the lowered temperature contributed to rVH binder acquisition by enhancing soluble expression^27^ of rVHs fused to gIIIp and/or suppressing thermal unfolding.

**Figure 4:**
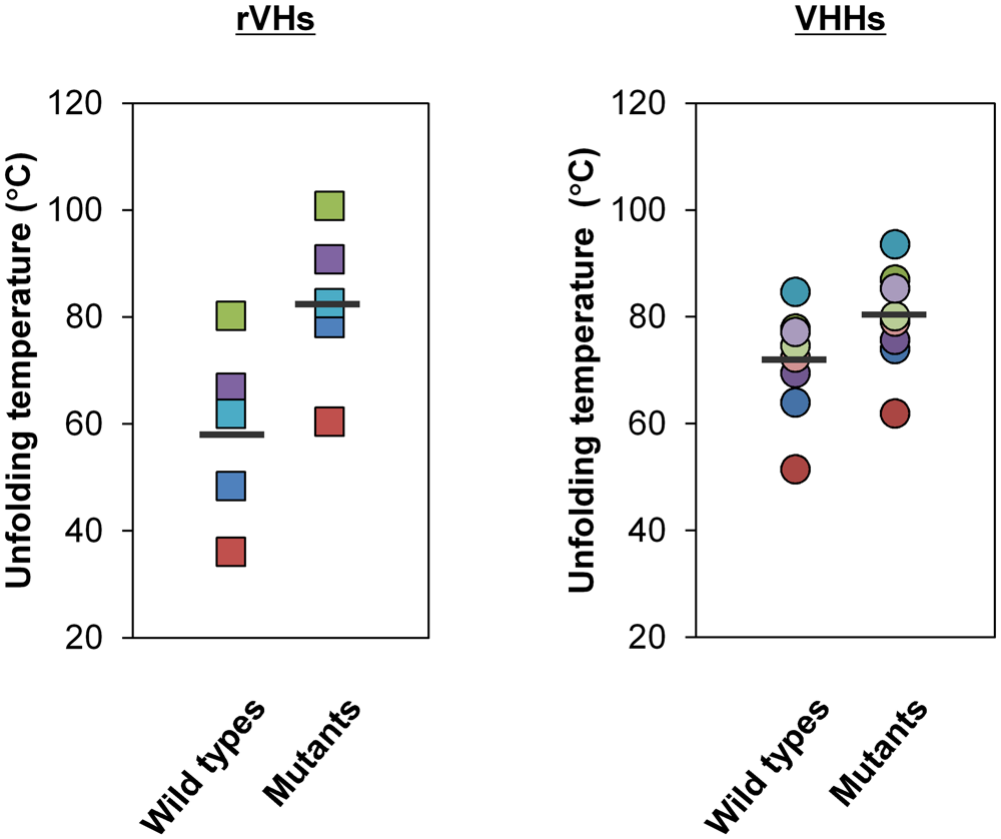
Comparison of increase in the unfolding temperature of rVHs and VHHs due to C54-C78 introduction. Unfolding temperature of wild types and their corresponding C54-C78 mutants are indicated as the same symbol and color for rVH and VHH. Average thermal unfolding temperatures of each group are indicated with bold lines (rVHs = 58.7 °C, mutant rVHs = 82.7 °C, VHHs = 71.9 °C, mutant VHHs = 80.9 °C). The unfolding temperatures of VHHs were quoted from refs 22–24.

For industrial applications as therapeutic agents, a simple and universal method is strongly needed to enhance the thermal stability of rVHs. This study showed that the introduction of C54-C78 increased unfolding temperatures of rVHs, and surprisingly their shifts of unfolding temperature were much larger than those of VHHs^22–24^ (24.0 °C for rVHs vs 9.0 °C for VHHs on average, Fig. 4). The thermal stabilities of mutant rVHs were comparable to those of mutant VHHs. We considered our approach is applicable not only to our representative rVHs but also any other rVHs because almost all of the rVH frameworks (80–90%) have high sequence similarity due to adopting only one germ-line gene segment^9, 11, 12^ and the beneficial mutations in the framework could be shared among the rVHs. To be employed for various applications, expression yield in *E. coli* might be one of the important factors for sdAbs. Improvement of purification yield was not clearly observed by disulfide bond introduction under our preparation conditions, however, in the case of H2-1-1, purification yield increased by 3- or 7-fold due to disulfide bond introduction when it was prepared using mammalian cell. This result supports the correlation between thermal stabilities and purification yields as suggested in Supplementary Figure S6. Besides the conformational stability, protein yield could be influenced by various factors including mRNA transcription and translation efficiency, folding efficiency, solubility, and so on. Therefore, by optimizing preparation conditions including modification of expression vectors and *E. coli* strains, introduction of disulfide bonds could increase purification yield using *E. coli* expression system.

We next examined the thermodynamic parameters to reveal the origin of the larger *T*_m_ increase in the C54-C78 mutant of H2-2-2 compared to the mutant of VHH_hCG_. The Δ*G* increase at each ${T}_{{\rm{m}}}^{{\rm{W}}}$ (ΔΔ*G*(${T}_{{\rm{m}}}^{{\rm{W}}}$)) accompanied by the mutation was 9.9 kJ/mol for H2-2-2 and 10.4 kJ/mol for VHH_hCG_, which were almost the same values as one another. Then, under the condition where ΔΔ*G*(${T}_{{\rm{m}}}^{{\rm{W}}}$) is a constant value, we investigated how the *T*_m_s of mutant (${T}_{{\rm{m}}}^{{\rm{W}}}$) changes when different values of other thermodynamic parameters are given. For simplification, we at first considered the case where Δ*C*_P_ is zero and then Δ*H* and Δ*S* are constant. Δ*G* of mutant is indicated by Equation 1.

1 $${\rm{\Delta }}G={\rm{\Delta }}H-T{\rm{\Delta }}S$$

Here Δ*G* at ${T}_{{\rm{m}}}^{{\rm{W}}}$ is expressed as Δ*G*°, while Δ*G* becomes zero at ${T}_{{\rm{m}}}^{{\rm{W}}}$, Δ*H* is indicated as Equations 2 and 3 from Equation 1.

2 $${\rm{\Delta }}H={\rm{\Delta }}G^\circ +{T}_{{\rm{m}}}^{{\rm{w}}}{\rm{\Delta }}S$$

3 $${\rm{\Delta }}H={T}_{{\rm{m}}}^{{\rm{M}}}{\rm{\Delta }}S$$

From Equations 2 and 3, ${T}_{{\rm{m}}}^{{\rm{M}}}$ can be indicated as Equation 4.

4 $${T}_{{\rm{m}}}^{{\rm{W}}}={T}_{{\rm{m}}}^{{\rm{W}}}+{\rm{\Delta }}G^\circ /{\rm{\Delta }}S$$

Thus,

5 $${\rm{\Delta }}{T}_{{\rm{m}}}={T}_{{\rm{m}}}^{{\rm{M}}}-{T}_{{\rm{m}}}^{{\rm{W}}}={\rm{\Delta }}{G}^{0}/{\rm{\Delta }}S$$

Equation 5 indicates that when Δ*G*° is unchanged, the smaller Δ*S* gives a larger Δ*T*_m_. In fact, when the Δ*S* of the mutant of VHH_hCG_ (0.96 kJ/mol/K) was employed instead of that of the mutant of H2-2-2 (0.36 kJ/mol/K), the calculated Δ*T*_m_ of the mutant of H2-2-2 decreased from 27 °C to 10 °C. Next, the Δ*G* of the mutant of H2-2-2 was calculated when its Δ*C*_P_ (1.8 kJ/mol) takes zero or Δ*C*_P_ of the mutant of VHH_hCG_ (5.4 kJ/mol), under the condition that Δ*G*, Δ*H* and Δ*S* at ${T}_{{\rm{m}}}^{{\rm{W}}}$ are unchanged (Supplementary Fig. S8). The smaller Δ*C*_P_ apparently resulted in the smaller Δ*G* temperature dependence, and Δ*C*_P_ of the mutant of H2-2-2 resulted in a Δ*T*_m_ of 23 °C, which is higher than the Δ*T*_m_ (19 °C) calculated using Δ*C*_P_ of the mutant of VHH_hCG_. These estimations indicated that the small Δ*S* and Δ*C*_P_ values of the mutant of H2-2-2 could be causes of its higher Δ*T*_m_.

As for the Δ*G* increase of the mutant of H2-2-2, Fig. 3d suggested the contribution of a large negative ΔΔ*S*. In order to have further thermodynamic insight into the effect of C54-C78 introduction to rVHs, we compared experimental ΔΔ*S* of H2-2-2 with its theoretical entropy change of unfolded state by C54-C78 formation (Δ*S*_calc_). In the classical chain-entropy model, an enhancement of stability by the disulfide bond formation is primarily considered to be attributed to Δ*S*_calc_^28–31^. The Δ*S*_calc_ can be calculated using an equation^28^:

6 $${\rm{\Delta }}{S}_{{\rm{calc}}}=-{\rm{R}}(3/2\,\mathrm{ln}\,{\rm{N}}+{\rm{A}})$$

where, R is the universal gas constant (8.31 J/mol/K) and N is the number of residues in the disulfide bond-linked loops (21 residues). Constant A was proposed by Poland and Scheraga^28^ and Pace *et al*.^29^ as 3.5 and 1.1, respectively. The Δ*S*_calc_ values were respectively calculated to −66.7 and −46.7 J/mol/K with each constant A. Both of the Δ*S*_calc_ values were far different from the experimentally obtained ΔΔ*S* (−154.4 J/mol/K at 35.0 °C), indicating that the classical chain-entropy model cannot be applied to rVH. Such a large discrepancy suggests that the restricted chain configuration of the unfolded state could not be the only effect of the C54-C78 introduction for H2-2-2. Changes in the internal interaction and/or hydration state might be other effects contributing to the mutant of H2-2-2 Δ*G* increase as reported for VHH_hCG_^22^.

In this study, we obtained rVHs without their partner rVLs by displaying only rVHs on the phage. Isolation of VH from Fv accompanies the exposure of the VL-interacting surface, which is generally composed of hydrophobic amino acids, to solvent. The exposure of the VL-interacting surface could cause intermolecular interactions and thereby decrease colloidal stability, leading to undesirable non-specific oligomerizations and eventually aggregations^26^. The features of VL-interacting surfaces of rVHs obtained in this study were next investigated and compared with those of rVHs that were previously obtained as Fv^32–35^. The amino acid residues located on this surface (cyan-colored in Fig. 2b) are listed in Supplementary Figure S9a. Comparing the ASAs of our rVHs and rVHs from Fv, no significant difference was found in the ASA of non-polar groups (ASA_non-pol_, Supplementary Fig. S9b). On the other hand, our rVHs showed a clear tendency of larger ASA polar groups (ASA_pol_) than rVHs in Fv. Surprisingly, this significant difference was mainly attributed to only one amino acid at the residue 120. Most of the obtained rVHs had Gln at residue 120 while all rVHs in Fv had Pro. Compared with the Pro at residue 120, Gln had a smaller ASA_non-pol_ (30 Å^2^–40 Å^2^ smaller) and larger ASA_pol_ (80 Å^2^–100 Å^2^ larger) (Supplementary Fig. S9c), providing a higher hydrophilic surface area at the VL-interacting surface of our rVHs. These results might originate from the elimination of rVHs with hydrophobic VL-interacting surfaces during the panning step using hydrophobic magnetic beads and microtubes. Considering that the VHHs, which are a natural single domain variable region of camelids, also adopt Gln^22–24^, Gln at the residue 120 of sdAbs might generally be advantageous from the point of colloidal stability. Further investigation of colloidal stabilities, three-dimensional structures of rVHs, and their molecular states in highly concentrated solution^36^ will clarify the general rules of VH stabilization.

In conclusion, rVHs proved to have sufficiently high affinities that could not be achieved by VH from mice and human IgGs. The low thermal stability concern of rVHs was eliminated by introducing an additional disulfide bond. Thus rVHs are a promising new source of sdAbs, and their higher availability than conventional sdAbs would enable more frequent usage of sdAbs in various applications as therapeutic use.

## Materials and Methods

### Rabbit immunization

Three Japanese white rabbits (Inoue-shouten, Takasaki-Shi, Gunma, Japan) were immunized with a mixture of 33 μg of recombinant human ErbB-2/HER2 protein (ACROBiosystems, Newark, DE, USA) and recombinant human ErbB-3/HER3 protein (ACROBiosystems) in combination with Freund’s Complete Adjuvant. Seven days after the first immunization, rabbits were re-immunized with the same antigen mixture with Freund’s Incomplete Adjuvant, and this process was repeated eight times every two weeks. Seven days after the final immunization, rabbits were euthanized to isolate spleen and lymph node cells. The serum titers of each antigen were checked by Enzyme-Linked ImmunoSorbent Assay (ELISA) using HRP conjugated goat anti-Rabbit antibody (Immuno-Biological Laboratories Co., Ltd., IBL, Fujioka-Shi, Gunma, Japan) at seven days after the fourth, sixth and last immunization, respectively. All experiments with animals were approved by the Institutional Animal Care and Use Committee of Daiichi Sankyo and carried out in strict accordance with the IBL guidelines for animal experiments, which complies with the laws concerning animal protection and management.

### Preparation of rVH-displaying phage library

From a total 1.9 × 10^8^ spleen and lymph node cells of immunized rabbits, mRNAs were extracted using Dynabeads mRNA DIRECT Kit (Life Technologies Corporation, Grand Island, NY, USA) and reverse transcribed to cDNA with Transcriptor High Fidelity cDNA Synthesis Kit (Roche, Basel, Switzerland). Four 5′-sense and two 3′-antisense primers were designed to cover all rVH germ-line sequences (Supplementary Table S1) and used for polymerase chain reaction (PCR) to amplify rVH genes from the synthesized cDNA library. Amplified rVH genes were inserted into the Sfi I/Not I site of phagemid vector pCANTAB5E (Amersham plc, Buckinghamshire, UK) and fused following the 5′ end of the E-tag (GAPVPYPDPLEPR) and the gIII coat protein of M13 bacteriophage (gIIIp) coding sequence. *E. coli* TG-1 strain (Agilent Technologies, La Jolla, CA, USA) was transformed with these phagemid vectors. Obtained transformants were pooled and infected with enough amounts of helper phage VCSM13 (multiplicity of infection >100). Subsequently rVH displaying phages were produced by cultivation of these transformants at 16, 20, 22 or 25 °C overnight with 2× YT medium supplemented with 0.25 mM IPTG, 100 μg/mL ampicillin, and 50 μg/mL kanamycin. Produced phage was then precipitated from overnight cultured medium using polyethylene glycol 6,000 and dissolved with phosphate buffered saline (PBS). Comparison of rVH display levels on the phage, 1.0 × 10^10^ virions of phages, produced at each temperature were subjected to western blotting (WB) using anti-E-tag antibody (Bethyl Laboratories, Montgomery, TX, USA), and appropriate secondary antibodies were used for detections. Virion numbers of purified phages were quantified using spectrophotometry with the following formula^37^.

$${\rm{Number}}\,{\rm{of}}\,\mathrm{virions}/\mathrm{mL}={({\rm{A}}}_{{\rm{269}}}-{{\rm{A}}}_{{\rm{320}}})\times {\rm{6}}\times {{\rm{10}}}^{{\rm{16}}}/(\mathrm{number}\,{\rm{of}}\,{\rm{nucleotide}}\,{\rm{bases}}/\mathrm{virion})$$

Here, A_269_ and A_320_ indicate UV absorption of 269 and 320 nm, respectively, and the number of nucleotide bases per virion was set to be 5000.

### Panning against antigens

The rVH displaying phage library was first subjected to negative selection using Dynabeads M-280 Streptavidin (Life Technologies Corporation) without antigen for eliminating non-specific binders. All beads were previously blocked with BSA (Jackson ImmunoResearch Laboratories, Inc., West Grove, PA, USA) in all of the experiments. rVH-displaying phages unbound to the beads were then exposed to 50 pmol of biotinylated HER2 or HER3 at 4 °C for overnight (first round) or at room temperature for one hour (second and third rounds). Biotinylated antigens were prepared using ChromaLink™ Biotin Antibody Labeling Kit (Solulink, Inc., San Diego, CA, USA) according to the manufacturer’s instructions. Subsequently, new beads were added to recover biotinylated antigen binders. Library treated beads were washed by three different conditions as follows: PBS containing 3% (w/v) BSA and 0.05% Tween-20, PBS with 0.05% Tween-20, and PBS, respectively. Specific binders were eluted with 0.1 M Glycine-HCl (pH 2.2) and immediately neutralized with 1 M Tris-HCl (pH 8.0). After that, *E. coli* TG-1 strain was infected with eluted phages and cultivated on an LB agar plate supplemented with 100 μg/mL of ampicillin. Appearing colonies were used for the next round of phage production or ELISA screening. Phage recovery rates of each round of panning were calculated as a ratio of input to output titer (colony forming unit for TG-1). To confirm the concentration of antigen specific binders, panning without antigen was also conducted as a negative control at the third round.

### ELISA screening of antigen binding rVHs

A randomly selected 317 output colonies from the third panning were inoculated to 2× YT medium supplemented with 100 μg/mL of ampicillin and 0.1% (w/v) glucose and cultivated at 37 °C overnight. After final concentration of 0.5 mM, IPTG was added to induce rVH expression, and cultivation started again at 16 °C. Lysozymes were then added to overnight culture and the mixture was transferred to a Nunc MaxiSorp flat-bottom 96-well plate (Thermo Fisher Scientific, Inc., Waltham, MA, USA) precoated with antigen (signal) or bovine serum albumin (noise). Bound rVHs were detected with HRP-conjugated anti-E-tag antibody (Bethyl laboratories) and >2.0 as a signal-to-noise ratio was set as the criterion for positive. Positive clones with repeatability were regarded as a hit.

### Preparation of rVHs

Genes encoding hit rVH clones were genetically linked with FLAG and His tag by PCR and inserted into pFLAG-CTS vector (Sigma-Aldrich, St. Louis, MO, USA) by homologous recombination. *E. coli* BL21 (DE3) strain (Merck Millipore, Darmstadt, Germany) was transformed with expression vector and obtained transformants were grown in LB medium supplemented with 100 μg/mL of ampicillin. When optical density at 600 nm reached to 1.0, rVH expression was induced by addition of IPTG at a final concentration of 1 mM and cultured at 16 °C for overnight. Infected cell and culture medium was separated by centrifugation and collected cell pellets were subjected to osmotic shock with 20 mM Tris-HCl pH 8.0 supplemented with 0.5 M sucrose and 0.1 mM EDTA. The osmotic shocked supernatant was mixed with the cultured medium, and rVH was affinity purified from this mixture by using Ni Sepharose excel (GE Healthcare UK Ltd., Little Chalfont, Buckinghamshire, England). Concentration dependent ELISA were conducted using affinity purified rVHs, and as for VHs for evaluations of binding affinity and thermal stability, affinity purified rVHs were further purified with gel filtration using a Superdex 75 10/300 GL with AKTA system (GE Healthcare UK Ltd). rVH of the clone, H2-1-1, was prepared using an Expi293F mammalian cell expression system (Life Technologies Corporation) according to the manufacturer’s instructions. Genes encoding H2-1-1 or its C54-C78 mutant with FLAG and His tags were sub-cloned into pcDNA3.1 vector for mammalian expression and expressed H2-1-1 rVH was purified similarly to rVHs using an *E. coli* expression system. The purities of finally purified rVH samples were confirmed by SDS-PAGE analysis and the protein concentrations were determined from the absorbance of 280 nm with the extinction coefficients which were calculated from amino acid sequences in Fig. 2a using Sednterp ver. 1.09 (University of New Hampshire, USA).

### Physico-chemical property analysis of rVHs

Binding activities of hit rVHs were evaluated by ELISA at concentrations of 16, 125 and 1000 nM of affinity purified rVH with HRP conjugated anti-FLAG M2 antibody (Sigma-Aldrich). Those that showed apparent differences in intensities between antigen and BSA were regarded as antigen specific rVHs. Binding affinities were measured at 25 °C by SPR using BIAcore T200 with a Series S Sensor Chip CM5 (GE Healthcare UK Ltd) coated directly with HER2 or indirectly by ErbB3 Fc Chimera (R&D Systems, Inc., Minneapolis, MN, USA) via Human Antibody Capture Kit (GE Healthcare UK Ltd). The kinetic parameters were determined by a 1:1 binding model of single cycle kinetics using BIAcore T200 Software. For H2-2-2 and its C54-C78 mutant, binding affinities were re-evaluated with BLI method using Octet RED 384 system (Pall ForteBio LLC, Fremont, CA, USA) with Dip and Read™ Streptavidin Biosensors (Pall ForteBio LLC) coated with biotinylated HER2.

To estimate the molecular state of rVHs, SEC-MALS analysis was conducted using a DAWN HELEOS II 8+ (Wyatt Technology Corp, USA, Santa Barbara, CA) with a Sepax Zenix-C SEC-300 column (Sepax Technologies, Inc., USA, Newark, DE).

Regarding thermodynamic analysis, DSC was conducted using a MicroCal VP-Capillary DSC (Malvern Instruments Ltd, Worcestershire, UK.) at a heating rate of 60 °C/h. To evaluate thermal stability, the *T*_peak_ value (temperature where heat capacity takes the maximal value) was determined with rVH samples at a concentration of 0.2 mg/mL in PBS using the software MicroCal Origin 7 (Malvern). To obtain detailed thermodynamic parameters, DSC analysis was conducted at 1.0 mg/mL. The Δ*H* values were estimated by the integration of endothermic heat accompanied by the unfolding. The *T*_m_ values were determined as the temperature at which the integration of endothermic heat is equal to half the area of Δ*H*. The enthalpy change (Δ*H*(*T*)), entropy change (Δ*S*(*T*)) and free energy change (Δ*G*(*T*)) are indicated as Equations 7–9.

7 $${\rm{\Delta }}H(T)={\rm{\Delta }}H+{\rm{\Delta }}{C}_{{\rm{P}}}(T-{T}_{{\rm{m}}})$$

8 $${\rm{\Delta }}S(T)={\rm{\Delta }}S+{\rm{\Delta }}{C}_{{\rm{P}}}\,\mathrm{ln}(T/{T}_{{\rm{m}}})$$

9 $${\rm{\Delta }}G(T)={\rm{\Delta }}H(T)-T{\rm{\Delta }}S(T)$$

From Equations 7–9, Δ*S* can be calculated as Δ*H*/*T*_m_ because Δ*G* becomes zero at *T*_m_. In this analysis, we assumed that Δ*C*_P_ was constant and determined as the difference between the baseline of folded and unfolded states in the DSC curve.

### Model structure construction and structural analysis

Model structures for obtained rVHs were constructed with the antibody structure prediction function of BioLuminate (Schrodinger, New York, NY). The PDB data, which were employed to construct the model structure, are listed in Supplementary Table S3. To argue the possibility of additional disulfide bond introduction to rVHs, model structures of rVHs with additional disulfide bonds were created using the function of Discovery Studio Ver. 4.0 (Accelrys, San Diego, CA, USA). Structural analysis of VHH_hCG_ was conducted using available PDB data (PDB ID: 1HCV). The ASA of residues for Cys mutation and estimated RMSD of Cα of residues in the framework were calculated using Discovery Studio Ver. 4.0. To estimate hydrophobicity of VL-interacting surfaces in obtained rVHs, the ASA_pol_ and ASA_non-pol_ of residues located at the VL-interacting surface were calculated using Discovery Studio Ver. 4.0. The residues of rVH in the VL interacting surface were defined as the common residues, which are less than 5 Å from its VL, in the rVH of available rabbit Fv structures (PDB ID: 4HBC, 4HT1, 4JO1, 4JO4, 4O4Y). The ASAs of these rVHs, which were originally obtained as Fv^32–35^, were also calculated with the three dimensional coordinates of the VH without the VL portion, which had been derived from the crystal structures of the Fv.

### Introduction of additional disulfide bonds to rVHs

For additional disulfide bond introduction, Gly or Ala and Ile at positions 54 and 78 of rVHs were both mutated to Cys by site-directed mutagenesis. Cys introduced mutants were prepared by the same methods as wild type rVHs, and disulfide formations were confirmed for H2-1-1, H3-9, and H3-15 as follows. rVHs and their mutants were treated with sequencing grade chymotrypsin (Roche, Basel, Switzerland) at 37 °C for 4 hours followed by addition of 10% formic acid, and then subjected to LC-MS analysis using a Waters Synapt G2S (Waters Corporation, Milford, MA, USA). Peptide identification was conducted with MassLynx Mass Spectrometry Software ver. 4.1 and BiopharmaLynx Software ver. 1.3 (Waters Corporation).

## Electronic supplementary material

Supplementary Information
