# Supplementary material for: Efficient generation of single domain antibodies with high affinities and enhanced thermal stabilities
Source: Sci Rep. 2017 Jul 19;7:5794. doi: 10.1038/s41598-017-06277-x (PMC5517631; doi:10.1038/s41598-017-06277-x)
Supplement: Supplementary file 1 — Supplementary Information [file 41598_2017_6277_MOESM1_ESM.pdf]

Supplementary Information

Efficient generation of single domain antibodies with high affinities and enhanced thermal stabilities

Naoya Shinozaki,[1] Ryuji Hashimoto,[1] Kiichi Fukui,[2] Susumu Uchiyama[3, 4 *]

[1]Modality Research Laboratories, R&D Division, Daiichi Sankyo Co., Ltd., Shinagawa R&D Center, 1-2-58 Hiromachi, Shinagawa-ku, Tokyo 140-8710, Japan
[2]Graduate School of Pharmaceutical Sciences, Osaka University, 1-6 Yamadaoka, Suita, Osaka 565-0871, Japan
[3]Department of Biotechnology, Graduate School of Engineering, Osaka University, 2-1 Yamadaoka, Suita, Osaka 565-0871, Japan
[4]Okazaki Institute for Integrative Bioscience, National Institutes of Natural Sciences, 5-1 Higashiyama, Myodaiji, Okazaki, Aichi 444-8787, Japan

*Corresponding author
Tel. +81-6879-4216, Fax: +81-6879-7442,
e-mail: suchi@bio.eng.osaka-u.ac.jp

Rabbits

↓ *Immunization with antigen mixture (HER2 and HER3)*

Spleen and lymph node cells from immunized rabbits

↓ *mRNA preparation, cDNA synthesis,*
rVH gene amplification and cloning into phagemid vector

rVH displaying phage (produced at 16°C)

↓ *Three rounds of panning against HER2 or HER3*

Panning output rVHs

↓ *ELISA screening (HER2:317, HER3:317)*

↓ *Sequence analysis (HER2:55, HER3:125)*

↓ *Assay for concentration dependent binding for antigen*
and selectivity over BSA (HER2:13, HER3:12)

Antigen specific rVHs *(HER2:8, HER3:8)*

↓ *Introduction of additional disulfide bond*

Thermally stabilized rVH

Supplementary Figure S1. Flowchart outlining the strategy to produce rVHs with high affinities and thermal stabilities. Numbers in parentheses indicate the number of rVH clones for HER2 and HER3 in each step, respectively.

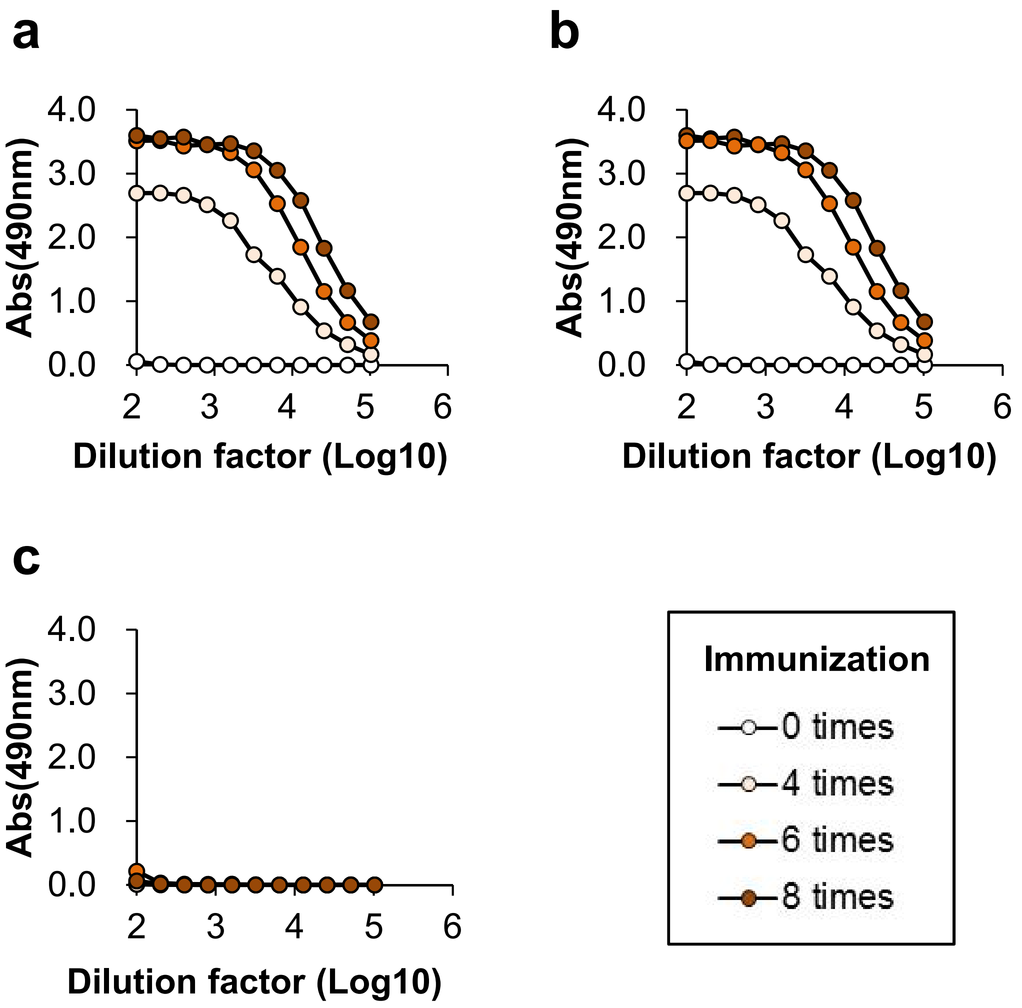

Supplementary Figure S2. Increase in the titers of rabbit serum due to immunization. Rabbit serum was collected before immunization and seven days after immunizations with the mixture of HER2 and HER3 and titrated against (a) HER2, (b) HER3, or (c) BSA.

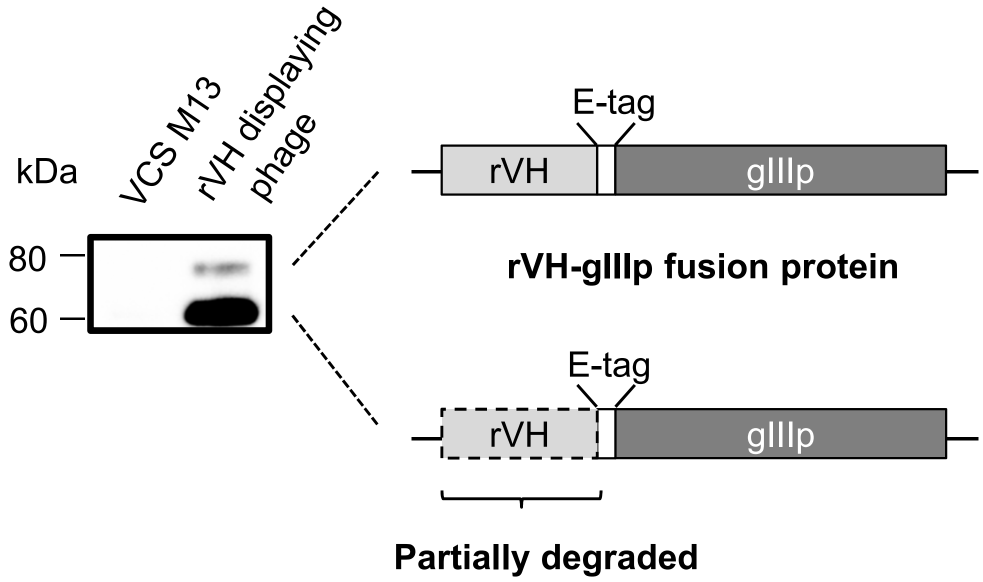

Supplementary Figure S3. Detection of rVHs displayed on the phage. The rVH displaying phage produced at 16°C and control phage VCS M13 (composed of only tag-free native gIIIp) were subjected to WB (1.0 ✕ 10^{10} virions per well). Detection of the rVH-gIIIp fusion protein was conducted with anti-E-tag antibody.

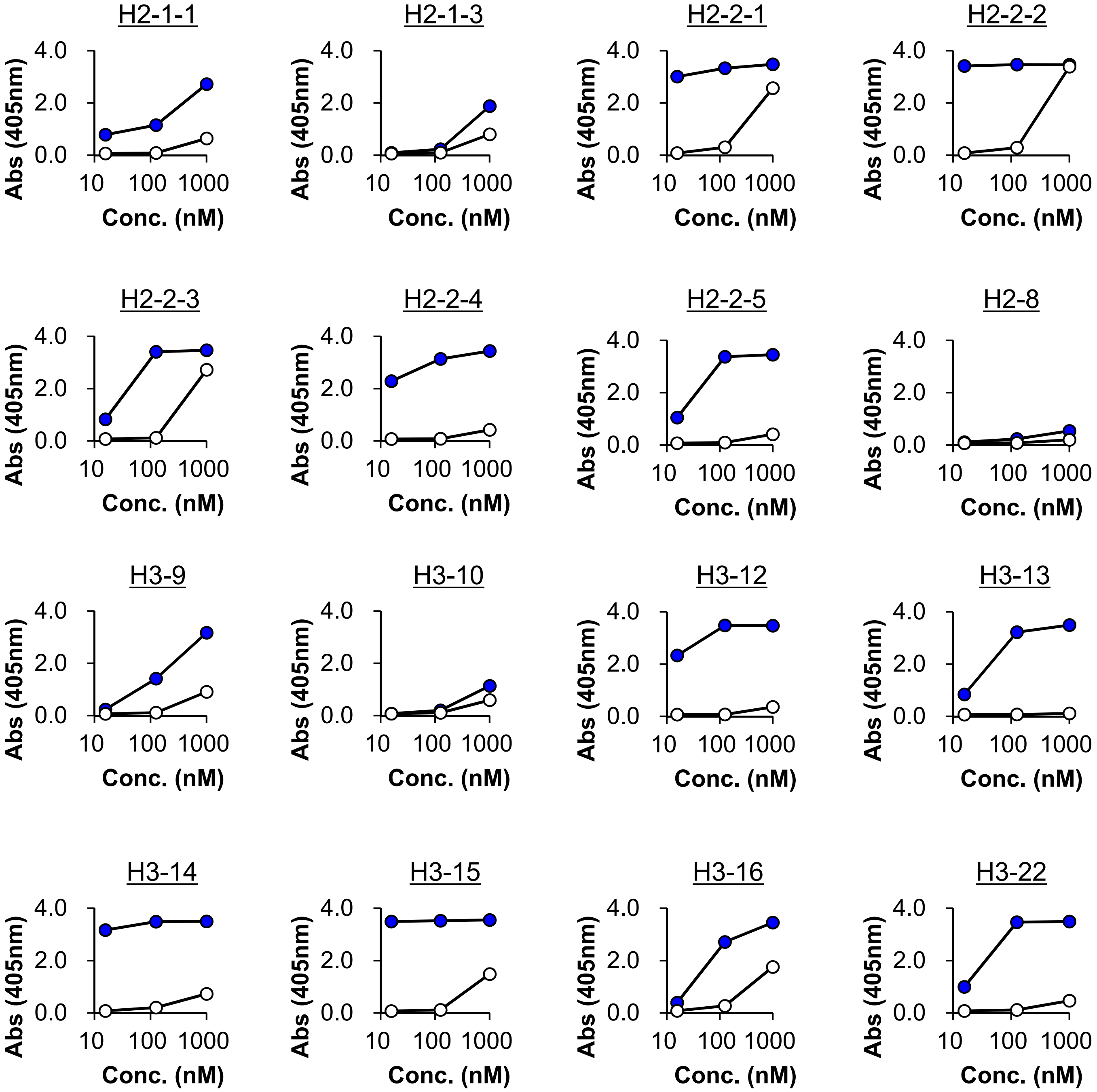

Supplementary Figure S4. Dose-dependent ELISA binding assay of hit rVHs. Binding of hit rVHs to HER2 or HER3 (Blue) or BSA (white) were evaluated at concentrations of 16, 150 and 1000 nM.

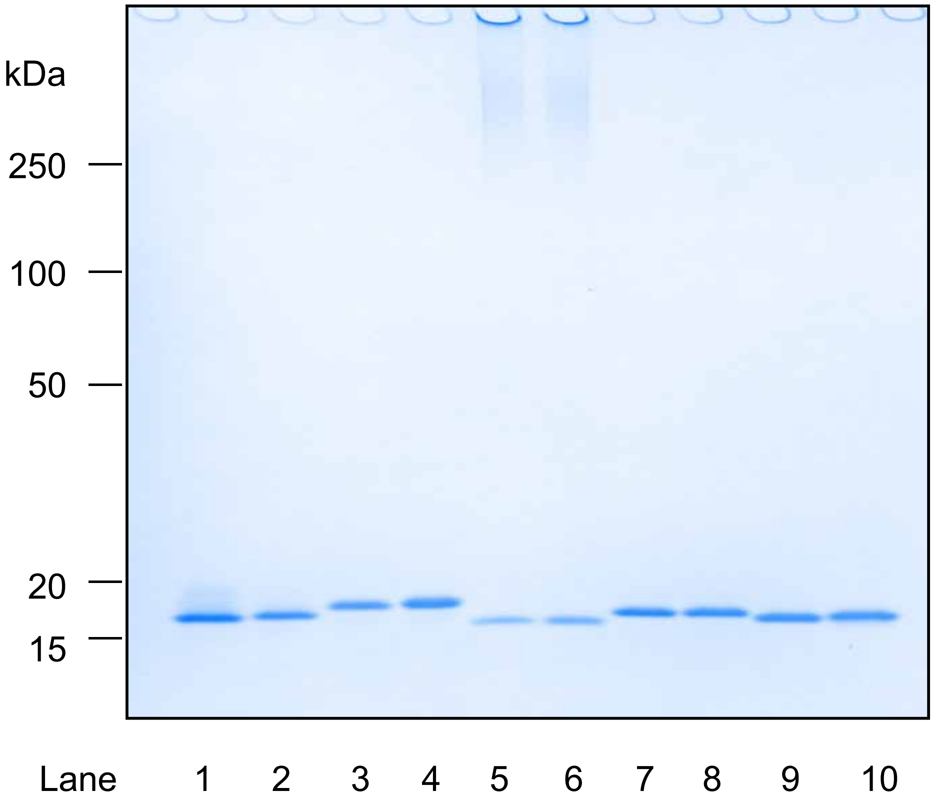

Supplementary Figure S5. Confirmation of the purities of rVHs used for physicochemical analysis. About 2 μg of each purified proteins were loaded. Lane 1, 3, 5, 7, 9 correspond to wild type H2-1-1, H2-2-2, H2-8, H3-9, and H3-15. Lane 2, 4, 6, 8, 10 correspond to C54-C78 mutants of H2-1-1, H2-2-2, H2-8, H3-9, and H3-15.

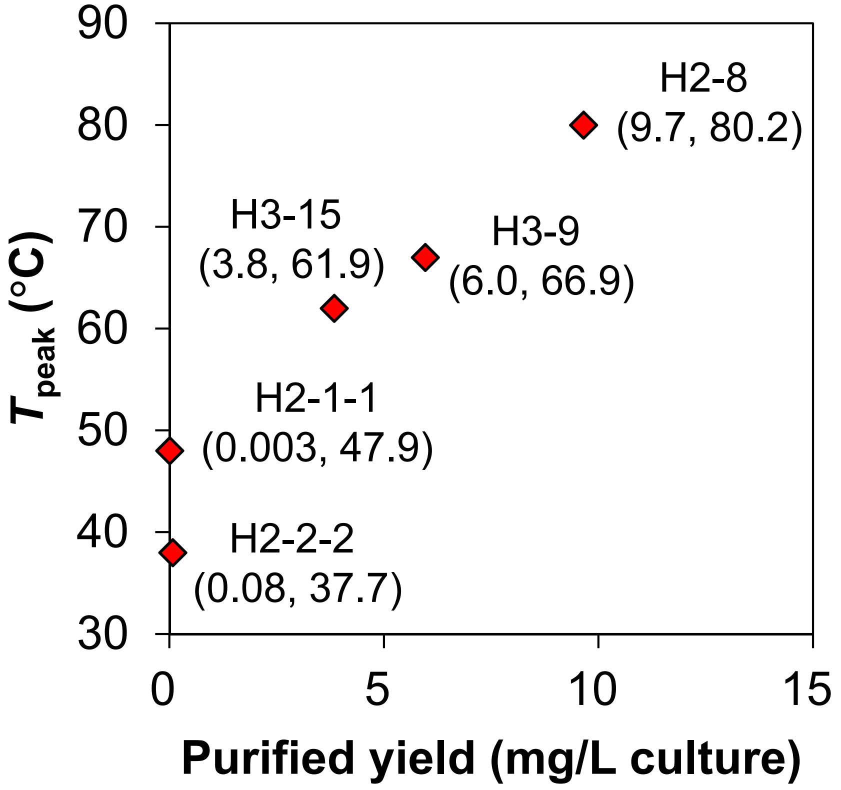

Supplementary Figure S6. Plot of the purification yield of wild type rVHs with *E.coli* expression system vs respective T_{peak} value. Figures in parenthesis indicate purification yield (mg/L culture) with *E.coli* expression system and T_{peak} value (°C) of each rVH.

a

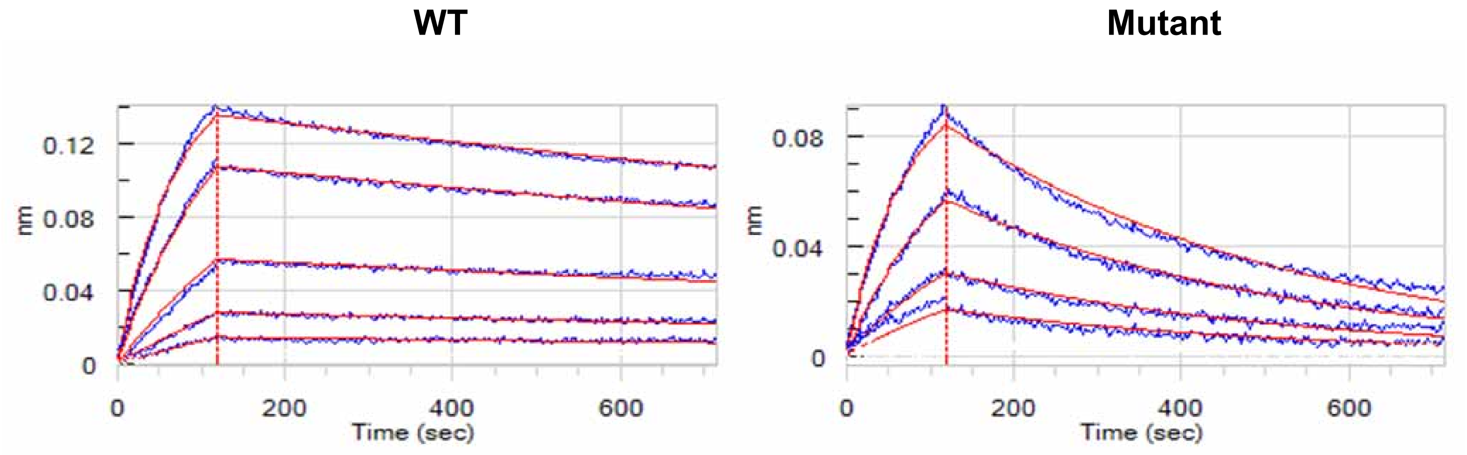

b

		k_{on} ($M^{-1}s^{-1}$)	k_{off} (s^{-1})	K_D (nM)	Change in K_D (fold)
SPR	WT	8.2×10^5	3.3×10^{-4}	0.4	
	Mutant	6.1×10^5	1.6×10^{-3}	2.7	6.8
BLI	WT	7.0×10^5	4.0×10^{-4}	0.6	
	Mutant	6.4×10^5	2.4×10^{-3}	3.8	6.3

Supplementary Figure S7. Comparing the changes in the K_D values due to disulfide bond introduction measured by SPR and BLI. (a) Observed sensorgrams (blue) and fitting curves (red) in the BLI analysis for H2-2-2. Data were collected at concentrations of 1.25, 2.5, 5, 10, 20 nM for H2-2-2 and 2.5, 5, 10, 20 nM for its C54-C78 mutant. (b) Obtained kinetic parameters of H2-2-2 WT and its mutant with SPR and BLI.

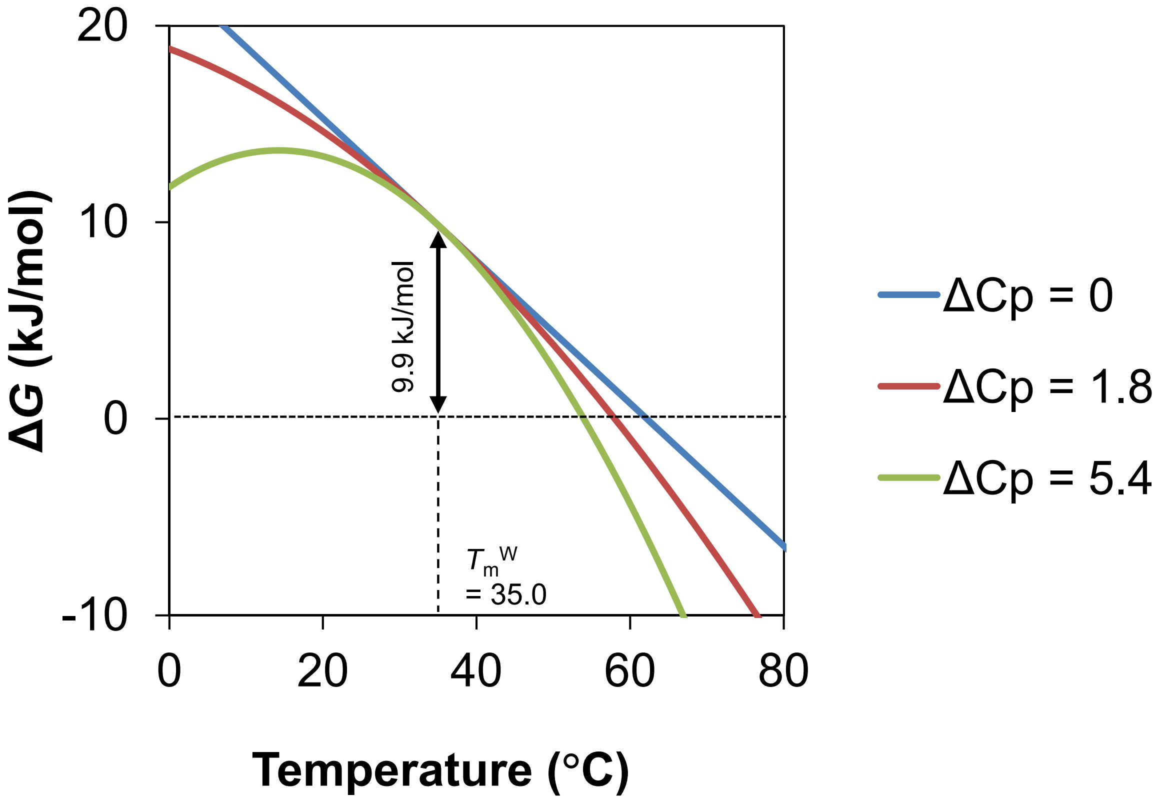

Supplementary Figure S8. Simulation of Δ*G*(*T*) for H2-2-2 with different ΔC_P. The simulated Δ*G*(*T*) are indicated for H2-2-2 with ΔC_P values of 0 (blue line), 1.8 (red line) and 5.4 (green line) under the condition that Δ*G*, Δ*H* and Δ*S* at T_m^W are unchanged.

a

		FR2							CDR2	FR3	FR4	
IMGT No.		42	44	48	49	50	51	52	65	103	118	**120**
rVH (w/o VL)	H2-1-1	V	Q	K	G	L	E	W	F	F	W	**Q**
	H2-2-2	V	Q	K	G	L	E	W	A	F	W	**Q**
	H2-8	V	Q	E	G	L	E	W	W	F	W	**Q**
	H3-9	V	Q	K	G	L	E	W	Y	F	W	**P**
	H3-10	V	Q	K	G	L	E	W	Y	F	W	**Q**
	H3-12	V	Q	K	G	L	E	W	Y	F	W	**P**
	H3-13	V	Q	K	G	L	E	W	Y	F	W	**P**
	H3-14	V	Q	K	G	L	E	Y	W	F	W	**Q**
	H3-15	V	Q	K	G	L	E	Y	Y	F	W	**Q**
	H3-16	V	Q	K	G	L	E	W	W	F	W	**Q**
	H3-22	V	Q	K	G	L	E	W	W	F	W	**Q**
rVH (w/ VL)	4HBC	V	Q	K	G	L	Q	W	Y	F	W	**P**
	4HT1	V	Q	K	G	L	E	W	Y	F	W	**P**
	4JO1	V	Q	K	G	L	E	W	A	F	W	**P**
	4JO4	V	Q	K	G	L	E	W	F	F	W	**P**
	4O4Y	V	Q	K	G	L	E	W	W	F	W	**P**

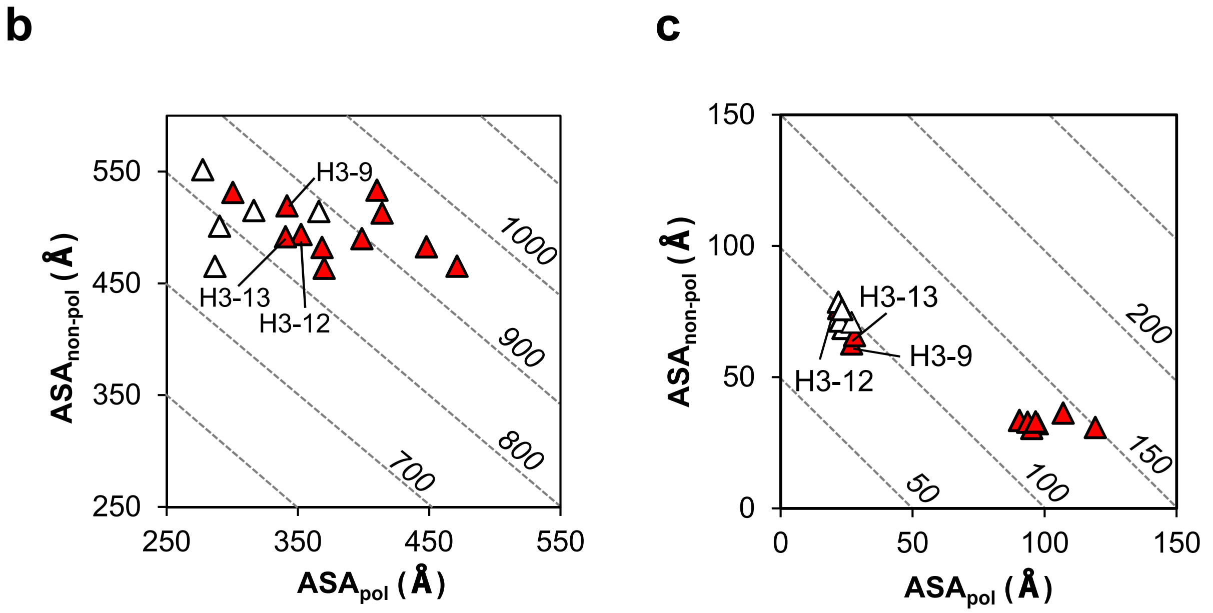

Supplementary Figure S9. Amino acids of VH-VL interacting surface of rVHs obtained with and without VL. (a) Amino acids of residues in the VH-VL interacting surface are indicated. Amino acids of (b) distinctive residue 120 are highlighted as bold.

Supplementary Table S1. Primers for amplification of rabbit VH genes

5’ sense primers
1, 5’-TAACAATA GGCCCAGCCGGCC CAGTCGBTGGAGGAGTCCGG-3’
2, 5’-TAACAATA GGCCCAGCCGGCC CAGTCRGTGAAGGAGTCCGAG-3’
3, 5’-TAACAATA GGCCCAGCCGGCC CAGTCGSTGGAGGAGTCCAGG-3’
4, 5’-TAACAATA GGCCCAGCCGGCC CAGGAGCAGCTGRWGGAGTCC -3’
3’ antisense primers
1, 5’-TTTTCCTTTT GCGGCCGC TGAAGAGAYGGTGACSAGGG-3’
2, 5’-TTTTCCTTTT GCGGCCGC TGARGAGACRGTGACCAGGG-3’

R = A or G; S = G or C; W = C or T; Y = C or T

Supplementary Table S2. Peptides linked by disulfide bond detected in MS analysis of rVH

		Detected peptides linked by disulfide bond		MW_{cal}	MW_{exp}	ΔMW
		Component 1	Component 2	(Da)	(Da)	(Da)
H2-1-1	WT	(L) TLTCTVSGF (S)	(F) CARDEARLPY (Y)	2117.99	2118.00	-0.01
		(L) TLTCTVSGF (S)	(Y) FCARDEARLPY (Y)	2265.06	2265.06	0
	Mutant	(L) TLTCTVSGF (S)	(F) CARDEARLPY (Y)	2117.99	2118.00	-0.01
		(L) TLTCTVSGF (S)	(Y) FCARDEARLPY (Y)	2265.06	2265.06	0
		(F) TCSKTSTTVDL (K)	(W) ICIM (S)	1630.76	1630.76	0
H3-9	WT	(L) TCTVSGF (S)	(F) CARGY (S)	1279.53	1279.54	-0.01
	Mutant	(L) TCTVSGF (S)	(F) CARGY (S)	1279.53	1279.54	-0.01
		(W) ICIIGPTDNTY (Y)	(F) TCSKTSSTTVDL (K)	2448.14	2448.14	0
H3-15	WT	(L) TCTASGF (S)	(F) CARIGSNSGW (G)	1732.73	1732.74	-0.01
	Mutant	(L) TCTASGF (S)	(F) CARIGSNSGW (G)	1732.73	1732.74	-0.01
		(Y) ICM (I)	(F) TCSKTSTTVEL (K)	1531.69	1531.72	-0.03

MW_{cal}: Molecular weight calculated from amino acid composition
MW_{exp}: Experimentally obtained molecular weight
ΔMW: Difference between MW_{exp} and MW_{cal}

Supplementary Table S3. Component structures used to construct model structures of obtained rVHs

	Framework	CDR1	CDR2	CDR3
H2-1-1	4HBC	3IY6	2DTM	1MCP
H2-2-2	4JO3	3QNZ	3H0T	1DFB
H2-8	4HBC	3IY6	3IY1	3N85
H3-9	4HBC	2GJZ	2F5A	2VXV
H3-15	4HBC	1IFH	3STB	3IY3

The PDB IDs of component structures of rabbit antibodies used to construct model structures of obtained rVHs are indicated.

Supplementary Table S4. The ASA of residues for Cys replacement and estimated root mean square deviation (RMSD) of Cα of residues in the framework were calculated using model structures of WT and its additional disulfide bond introduced variant

	ASA (%)		RMSD (Å)
	G/A54	I79	
H2-1-1	0	7.4	0.3
H2-2-2	0	5.0	0.2
H2-8	0	8.6	0.3
H3-9	0	8.6	0.3
H3-15	0	8.6	0.4
VHH_{hCG}	3.8	5.6	0.3
